# Organic management pattern improves microbial community diversity and alters microbial network structure in karst tea plantation

**DOI:** 10.1016/j.heliyon.2024.e31528

**Published:** 2024-05-19

**Authors:** Tianyi Pu, Ni Zhang, Jinqiu Wang, Zhibing Zhao, Weiwen Tan, Can Li, Yuehua Song

**Affiliations:** a School of Karst Science, Guizhou Normal University/State Engineering Technology Institute for Karst Desertification Control, Guiyang, 550001, China; b Guizhou Provincial Key Laboratory for Rare Animal and Economic Insect of the Mountainous Region, Guiyang University, Guiyang, Guizhou, 550001, China

**Keywords:** Tea quality, Soil fertility, Soil microbial network analysis, Metagenome, Different management patterns, Karst

## Abstract

Soil microbiomes play a crucial role in enhancing plant growth, health, and overall agricultural productivity. Nevertheless, the influence of distinct agricultural management practices on the microbial diversity and community structure within tea (*Camellia sinensis*) plantations has remained enigmatic. This study postulates that organic agricultural management models can enhance microbial diversity and optimise the microbial community structure within tea plantations, indirectly augmenting soil fertility and tea quality. We employed metagenome technology and conducted molecular ecological network analysis to explore the impact of organic management, pollution-free management, and conventional management on the microbial network structure of tea plantation soil in Weng'an County in the southwestern karst region. Soils subjected to organic management exhibited a higher relative abundance of soil microbial and carbohydrate-active enzyme functional genes than those subjected to other management regimes. Additionally, the relative abundance and diversity of dominant bacteria and keystone species were notably higher under organic management than under the other management regimes. Correlation analysis showed that soil microorganisms were closely related to soil fertility and tea quality, respectively. One-way analysis of variance and the structural equation modelling results showed significant variability in soil fertility under the three agricultural management modes and that soil fertility and soil microbial diversity had a direct impact on tea quality (*P* > 0.05). In conclusion, this study underscores the profound impact of management modes on microbial diversity and community structure within tea plantations. These management practices alter the soil microbial network structure and potential function, ultimately regulating the microecological dynamics of the soil community in tea plantations.

## Highlights

• Agricultural management practices affect soil nutrients in tea plantations significantly.
• Organic management optimizes soil microbial community structure and microbial functional genes in tea plantations.
• Organic management improves tea quality directly.

## 1 Introduction

Tea tree (*Camellia sinensis*) is a perennial evergreen plant, mainly distributed in tropical, subtropical, and temperate regions in 47 countries worldwide. It has a long history of cultivation as an essential cash crop in southern China [1]. In recent years, the scale of tea cultivation has been expanding, and its scale in China accounts for approximately 63 % of the global tea plantation area and 50 % of global tea production (http://www.gov.cn/). Different agricultural management practices exert varying effects on soil health and plant growth [2]. Agricultural management encompasses aspects namely tillage, crop rotation, fertilisation, and pesticide use [3]. Long-term over fertilisation has been observed to negatively impact soil physicochemical properties and tea trees in tea plantations. Negative impacts include a decrease in soil pH, accumulation of aluminium (Al) and fluorine, nutrient leaching, and soil structure deterioration, all of which degrade tea quality [4,5]. Organic fertiliser application has emerged as a key practice that enhances the soil microbial environment, increases soil microbial activity, and promotes nutrient uptake and utilisation by crops, with enhanced crop yield and quality [[6], [7], [8]]. Therefore, the use of organic fertilisers instead of chemical fertilisers is one of the major organic farming strategies in tea plantations and improves tea leaf yield and quality [9].

Guizhou is a region with extremely developed karst landscapes, and its ecosystems are highly sensitive and vulnerable to environmental changes [10]. Planting tea trees is the main method for developing ecological agriculture in the mountainous areas of Guizhou. This type of intentional vegetation is also a crucial measure to restore the karst ecological environment [11] and provides ecological and economic benefits to the karst areas in Guizhou. Additionally, due to the influence of the soil-forming parent material in the karst region, the rate of rock weathering into the soil is slow, and the water-holding capacity is weak; thus, exploring the scientific and reasonable planting and management mode of tea plantation for the two-way optimisation of vegetation and soil is important [12].

The generally high background values of trace elements in Guizhou soils [13], which are released from rocks to soils through weathering and leaching processes [14], can affect the properties and microbial community of soil. The literature has found that microbial diversity was determined by soil properties and that microbial composition was driven by plant and soil properties [15], showing that the relationship between plants and soil is mutual [16]. Therefore, soil micronutrient content will also affect plant growth. However, few studies have investigated the relationship between plants and soils in karst areas. Soil microorganisms play integral roles in agroecosystems [17]. As regulators of biogeochemical cycles, microorganisms exert considerable influence on crop health and soil health [18]. In agroecosystems, land use intensity and agricultural practices are reportedly the key anthropogenic factors influencing the diversity and functional composition of soil microbial communities [3,19]. Notably, different fertilisation treatments influence microbial community structure in agricultural soils, affecting microbial functional genes [17]. Moreover, organic fertilisers and manure have been demonstrated to increase microbial diversity and alter microbial community composition significantly [20]. Chowdhury [21] demonstrated that long-term fertilisation practices (organic vs mineral) not only affected soil microbiota but also influenced plant-microbe interactions, influencing plant growth and health. One study has shown that organic fertilisers, the cornerstone of organic agriculture, optimise the microbial community structure and increase the abundance and diversity of bacteria and fungi in tea plantation soils [22]. Few studies have investigated the agricultural management patterns affecting the abundance, structure, and functional genes of soil microbial communities in tea plantations in karst areas.

Environmental microorganism macrogenomics involves targeted bioinformatics analysis based on genomic libraries of all the microorganisms present in an environment. The approach overcomes the limitations of traditional culturing methods, which may not capture certain microorganisms in the environment [23]. Sun and Badgley [24] employed metagenomics technology to study changes in soil microbial community function during forest ecological restoration and provided insights into the role of soil microorganisms in ecosystem function restoration. Microbial network analysis is a powerful tool for exploring microbiome metagenomic datasets, providing a comprehensive understanding of the intricate structure of soil biodiversity [25]. Gu [22] used network analysis to identify key species that contribute to microbial community composition and function, which indirectly affect soil fertility and crop yield [26]. However, the understanding of the use of metagenomics techniques to study the response of microbial communities and ecosystem functions in mountain tea plantations in karst areas to different agricultural management models remains poor.

Because of the imperative to fulfil the requirements of modern agricultural management models and sustainable agricultural development, this study used macroeconomics and network analysis to assess tea plantations managed by implementing three agricultural practices (organic [Organic, HY], pollution-free [PolFree, TS], and conventional management [Conv, XY]). The results provide fundamental theoretical support for the optimisation of management strategies and establishment of optimal tea-growing environments in mountainous tea plantations in the karst mountains, which is of substantial significance for improving the quality of tea and promoting the sustainable development of the tea industry. The authors hypothesised that (1) agricultural management practices affect soil nutrients in tea plantations significantly; (2) organic management optimizes soil microbial community structure and microbial functional genes in tea plantations; and (3) organic management improves tea quality directly.

## 2 Materials and methods

### 2.1 Study area

**Fig. 1:**
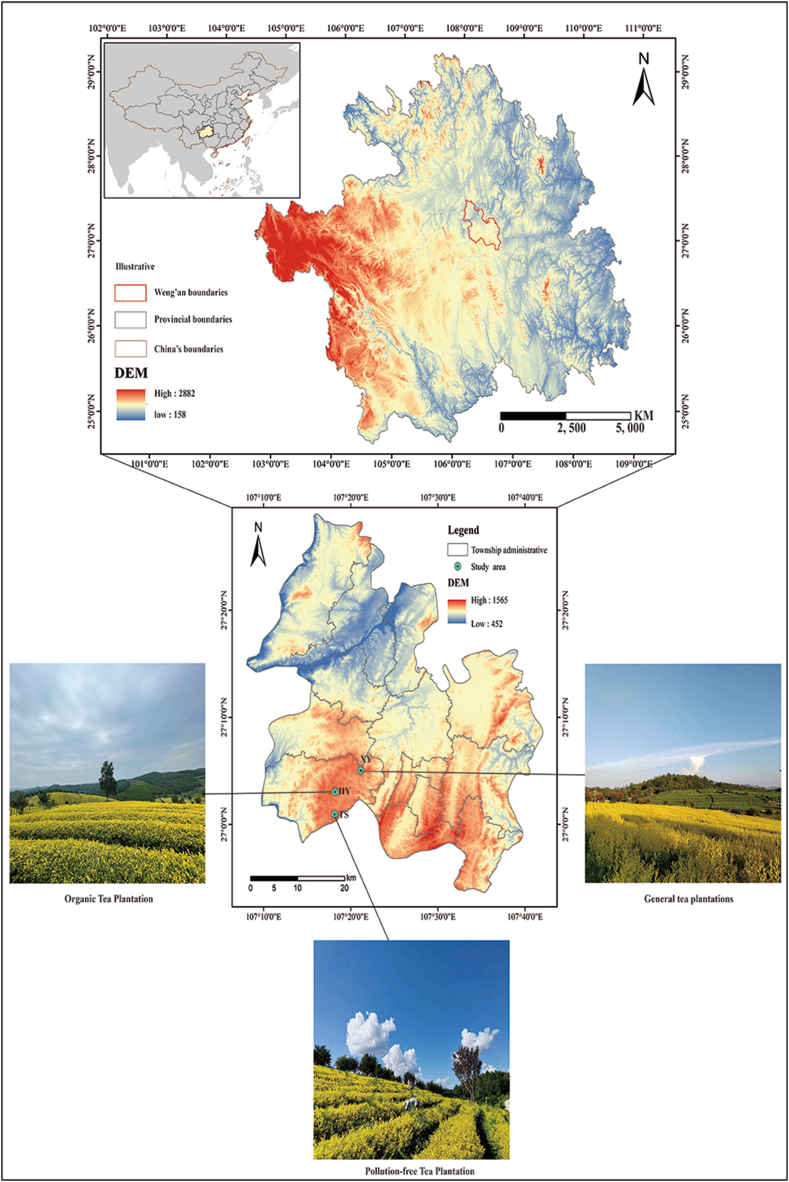
Location map of the study areas.

Weng'an County is in the middle of Guizhou Province, in the middle reaches of the Wujiang River, in the northernmost part of the Autonomous Prefectures Qiannan Buyei and Miao (107°28′18″ E, 27°04′12″ N). It has a subtropical humid monsoon climate, with an average temperature of 13.9 °C and an average precipitation of 1100–1150 mm, which is slightly acidic and suitable for tea tree growth. The soil is mainly yellow soil developed from sand shale. Additionally, as the largest centralised, continual planting base of ‘Huangjinya’ in China, Weng'an County is the main production area of European standard tea. ‘Huangjinya’ is a bright yellow tea with a bright yellow soup colour and pure yellow leaf bottom. It has a high amino acid content and a fresh taste favoured by tea consumers, and its cultivation has resulted in economic benefits for the area [27]. In 2018, because of its excellent quality traits, ‘Huangjinya’ became a protected geographical indication product and has been conferred a geographical indication certification trademark. In this study, Huang Hongyin (Organic, HY), Tianshun (PolFree, TS), and Xinyu (Conv, XY) tea plantations were selected as the study sites (Fig. 1). At the same time, the three tea plantations have been managed in the prescribed manner from the time the ‘Huangjinya’ tea trees were planted, with no change in management pattern in between. All study sites were planted with mature (>10 years) ‘Huangjinya’ tea trees.

### 2.2 Sample plot setting

This study aimed to investigate the effects on soil fertility and microbial community structure in tea plantations under different management practices and reveal the factors influencing the variation in tea quality across different agricultural management activities. To achieve these objectives, this study selected three agricultural management models—organic (HY), pollution-free (TS), and conventional (XY)—within the study sites in Weng'an County. The distances among the study sites were from 6.5 to 14 km; therefore, they had similar climatic backgrounds and geological conditions. The study sites were cultivated with the same variety and age (>10 years) of tea tree-'Huangjinya’; thus, they were influenced by similar natural factors. The characteristics of the sampling plots are shown in Table 1. As a pest control measure in the HY study site, tea was picked several times in batches in spring, deep tillage base fertiliser was applied in autumn, solar insect traps were installed every 30 m, and 150 g/666.7 m^2^ compound essential oil with 30 kg water spray was used 2–3 times per year. In the TS study site, regular weeding was performed, with deep ploughing in the autumn to apply basal fertiliser, and solar-powered insect-trapping lamps were installed at 30 m intervals. In the XY study site, regular weeding was performed, tea picking was infrequent, and deep ploughing was performed in the autumn to apply basal fertiliser to control pests. Therefore, the effects of different anthropogenic disturbances vary across the study sites.

**Table 1:** Study area information sheet.

Study Area	Fertilizer	Fertiliser Application Rate/Year	Cropping patterns	Longitude	Latitude	Age of Tree (year)	Row spacing/plant spacing (cm)	Altitude (m)	Growing Area (ha)
HY	Basal fertiliser + Bio-organic fertiliser (animal manure and urine) + Rapeseed cake	650–850 kg/666.7 m^2^	Compound planting pattern (tea tree + fruit tree)	107° 30 ‘31.76″ E	27° 05 ‘04.21'′ N	12	100-120/20	1315	120
TS	Base fertiliser + Tea fertiliser + Urea	250–350 kg/666.7 m^2^	Composite planting pattern (tea tree + ferns)	107° 30 ‘22.87″ E	27° 01 ‘55.56'′ N	10	100-110/17	1304	1.13
XY	Base fertiliser + organic fertiliser	200–240 kg/666.7 m^2^	Monoculture model	107° 35 ‘23.26″ E	27° 08 ‘40.30'′ N	8	90-100/13	1228	0.93

### 2.3 Sample collection

Soil samples were collected between 9 and August 10, 2022. There was sunny weather for more than 15 days prior to sampling, which ensured a low level of soil variability and a relatively low number of tests. For each sampling plot, 10 m × 10 m sample plots (with a sufficient buffer zone between plots) were established with 20 cm separation between plots. Multiple sampling points were laid along S-shaped lines in the sample squares, and at each sample point, equal amounts of soil obtained from 0 to 20 cm soil layers (less than 20 cm to the actual depth), mixed evenly, were collected. This method was used because 20 cm depth is the main depth at which the root system of the tea tree is concentrated. Therefore, after removing the humus layer, only the top 20 cm was sampled, and 25 soil samples were collected from each tea plantation. After the soil samples were cleared of stones and debris, the samples were stacked according to the diagonal method until the sample weight reached the value required to establish a composite sample. The collected samples were divided into two parts: one was used to determine their physicochemical properties, and the other was used for molecular experiments (sealed in a refrigerator at −80 °C for DNA extraction). Plant sample collection was conducted on August 19, 2022 (with sunny weather for more than 15 days prior to collection). Fifteen fresh leaf samples were collected from three tea plantations based on the standard of one bud and two leaves. The fresh leaves were steam-killed, roasted to dryness at 80 °C, and crushed to determine tea quality indicators.

### 2.4 Determination of physical and chemical properties of soils

Soil organic carbon (C; SOC) was determined using the potassium dichromate external heating method. The molybdenum-antimony anti-colorimetric method was used to determine total and available phosphorus (TP, AP). Total and available nitrogen (TN, AN) were determined using an automatic Kjeldahl apparatus (Shandong Haineng Scientific Instrument, Shandong, China). The ammonium acetate-flame photometry method was used to determine the available potassium (AK), and soil ammonia nitrogen (V) was determined using the hypochlorite-indophenol blue colourimetry. The analysis was performed using a soil agrochemical analysis method [28]. Soil bulk density (SBD) and water content (SWC) were determined using the ring knife method [29]. Soil pH was determined using the electrode potential method, and the water-soil ratio was determined by mixing at water and soil a ratio of 2.5:1 and allowing the mixture to stand for 30 min for extraction and determination.

The air-dried soil samples to be tested were mixed thoroughly; reduced to 100 g according to the tetrad method; crushed; passed through 0.435 and 0.149-mm aperture sieves, respectively; and loaded into different sample bottles for the testing and analysing of soil Zn, K, and Se, respectively. Soil metal elements (Zn, K) were determined directly by ICP-OES (iCAP-7200, Thermo Fisher Scientific, MA, USA) [30]. Soil Se was determined using AFS according to the national agricultural standard NY/T 1104–2006.)

### 2.5 Determination of tea quality properties

A sample of 0.2 g dried tea leaf powder was infused with 20 mL boiling distilled water in a boiling water bath for 45 min with manual shaking every 10 min. The tea infusion was then ﬁltrated using double-layer ﬁlter paper, and the volume was ﬁxed to 50 mL with distilled water after fully cooling the liquid at room temperature for further analysis. Tea polyphenol (GTP) was determined spectrophotometrically according to T/AHFIA 005–2018 (total polyphenol content in plant extracts and their products). The contents of WE were determined according to the national standard GB/T 8305-2013 [31]. The caffeine content was determined according to the national standard GB 5009.139–2014 [32]. Tea trace elements (Fe, Mn, Zn) were determined according to the national standard GB 5009.268–2016.)

### 2.6 DNA extraction and determination of soil microbial metagenomic amplicons

Total genomic DNA was extracted from fresh soil samples (0.5 g) by using the E.Z.N.A.® Soil DNA Kit (Omega Bio-tek, Norcross, GA, US) according to the manufacturer's instructions. The concentration and purity of the extracted DNA were determined using a TBS-380 fluorometer and a NanoDrop 2000 spectrophotometer, respectively. DNA extract quality was evaluated using 1 % agarose gel. The DNA extract was fragmented to an average size of approximately 400 bp by using a Covaris M220 (Gene Company Limited, China) for paired-end library construction. A paired-end library was also constructed using NEXTFLEX Rapid DNA-Seq (Bioo Scientific, Austin, TX, USA). Adapters containing the full complement of the sequencing primer hybridisation sites were ligated into the blunt ends of the fragments. Paired-end sequencing was performed on an Illumina NovaSeq/Hiseq Xten sequencing platform (Illumina Inc., San Diego, CA, USA) at Majorbio Bio-Pharm Technology Co., Ltd. (Shanghai, China) by using NovaSeq Reagent Kits/HiSeq X Reagent Kits according to the manufacturer's instructions (www.illumina.com). The data were analysed on the free online Majorbio Cloud Platform (www.majorbio.com). Paired-end Illumina reads were trimmed from adaptors, and low-quality reads (length <50 bp, quality value < 20, or having N bases) were removed using fastp (https://github.com/OpenGene/fastp, version 0.20.0). The metagenomic data were assembled using MEGAHIT (https://github.com/voutcn/megahit, version 1.1.2), which uses succinct de Bruijn graphs. Contigs with a length ≥300 bp were selected as the final assembly results and used for further gene prediction and annotation.

### 2.7 Soil microbial metagenome data processing and species and function annotation

Fastp [33] (https://github.com/OpenGene/fastp, version 0.20.0) was used to cut the adapter sequences at the 3′ and 5′ ends of reads, and high-quality pair-end reads and single-end reads were retained. MEGAHIT [34] (version 1.1.2) was used to assemble optimised sequences. Contigs ≥300 bp were selected as the final assembly results. Prodigal [35] and MetaGene [36] (http://metagene.cb.k.u-tokyo.ac.jp/) were used to predict the ORFs of contigs. CD-HIT [37] (http://www.bioinformatics.org/cd-hit/, version 4.6.1) was used to cluster the gene sequences predicted for all samples (parameters: 90 % identity, 90 % coverage). The longest gene in each class was used as a representative sequence to construct a non-redundant gene set. The high-quality reads and non-redundant gene sets of each sample were compared using SOA Paligner [38] software (http://soap. genomics. org. cm/, version 2.21). Carbohydrate-active enzyme annotation was conducted using hmmscan (http://hmmer.janelia.org/search/hmmscan) against the CAZy database (http://www.cazy.org/) with an e-value cutoff of 1e-5.

The WGCNA package was used to construct a co-occurrence network based on Spearman's correlation metrics of the soil bacteria, fungi, and archaea community [34]. Bacterial, fungal, and archaeal operational taxonomic units (OTUs) with an average relative abundance <0.01 % were removed from each OTU table. OTUs with zero relative abundance were also removed. The corAndPvalue function was used to calculate the microbial correlation cutoff and significance P. The Benjamini and Hochberg false discovery rate (FDR) corrects the p value [39]. A similarity threshold St of 0.50 was set when building the network. The correlation cutoff for the bacterial network construction was 0.5 with a P = 0.02, the fungal correlation cutoff score was 0.50 with a P = 0.05, and the archaeal cutoff score was 0.68 with a P = 0.05. Network clusters were identified using the igraph package, and subnetworks were extracted by preserving the phylotypes of individual soil samples using the induced subgraph function in the igraph package [40]. The network topology values were calculated using the calNetModule function, and Zi-Pi analysis, based on Node connectivity, was established based on among-module connectivity (Pi) and within-module connectivity (Zi), establishing four sub-categories of nodes (a) peripheral (Zi < 2.5; Pi < 0.62), (b) connectors (Zi < 2.5; Pi > 0.62), (c) module hubs (Zi > 2.5; Pi < 0.62), and (d) network hubs (Zi > 2.5; Pi > 0.62) [41]. The network was drawn using Gephi 0.9.2 [42].

### 2.8 Statistical analysis

One-way analysis of variance (ANOVA) was employed to assess differences in tea quality and soil physicochemical properties among tea plantations managed under different practices, followed by the least significant difference (LSD) test (P < 0.05). The data were log-transformed when necessary to achieve homogeneity of variances. The composition and diversity of soil microbial communities in tea plantations were analysed. Additionally, the relationships among tea quality factors, soil physicochemical properties, and microbial community structure were explored. This study analysed the effects of different agricultural management practices on microbial community structure based on the non-metric multidimensional scaling (NMDS) of the Bray–Curtis difference, combined with permutation multivariate ANOVA (PERMANOVA). Soil and tea factors were analysed using variance inflation factor (VIF), with a VIF <5, and redundancy analysis (RDA) and Mantel test analysis were performed. The statistical analyses were conducted using R software packages “vegan” [43] and “ggcor” [44]. Data visualization was performed using Origin 2021 (OriginLab, Northampton, MA, USA). Finally, segmented structural equation modelling (SEM) analysis was conducted using the R package “lavaan” [45] to assess the relationships among soil microbial community structure, soil physicochemical properties, and tea quality in tea plantations under different agricultural management practices. SEM model fit was described using p values (P > 0.05), fitted values (GIF >0.90), comparative fit indices (CIF >0.95), and the root mean square of the approximation error (RMSE <0.08).

## 3 Results

### 3.1 Effects of different agricultural management patterns on physicochemical properties and tea quality of tea plantation soil

ANOVA was used to test the responses of soil physicochemical factors to agricultural management treatment (Table S1). Excluding AK, NH_4_^+^-N, and SBD, the other soil physicochemical properties differed significantly under different agricultural management treatments (*P* < 0.05). The pH of the three tea plantations was within the 4.5–5.5 range, which is typically observed in high-yielding and high-quality tea plantations. The TP, Se, K, Zn, AL, AN, and AP content in the soil of the tea plantations revealed similar trends: HY > TS > XY (*P* < 0.05). Additionally, the TN and total organic C (TOC) contents in TS were higher than those in HY and XY.

WE, tea polyphenols (GTP), caffeine, and tea trace elements (Se, Fe, Mn, and Zn) are comprehensive indicators of the intrinsic chemical qualities of tea. In this study, ANOVA revealed significant differences in tea quality indicators (*P* < 0.05), excluding the Zn and WE concentrations (Table S1). The Mn, GTP, and caffeine contents in HY were significantly higher than those in TS and XY. In the tea, the Se content from HY was approximately three times higher than that from XY, and the Fe content from XY was 1.14 and 1.13 times higher than that from HY and TS, respectively (*P* < 0.05). Therefore, the Organic management practices resulted in higher Se, Mn, and Zn contents in tea leaves than under PolFree and Conv, the other two farming practices (at TS and XY, respectively).

### 3.2 Effects of agricultural management patterns on soil microbial communities in tea plantations

#### 3.2.1 Soil microbial community composition

The Illumina high-throughput sequencing platform was used to determine the microbial composition of all soil samples by using metagenomic sequencing technology. In total, 778,826,684 raw reads were generated from the 15 soil samples (an average of 51,921,779 reads per sample). The sample sequence was compared with the sequence in the NCBI non-redundant (NR) database to obtain species abundance data, which comprised 4,070,227 bacterial, 1728 fungal, and 25,120 archaeal genes. Using NR annotation, 161 phyla and 27,316 species of bacteria, 10 phyla and 549 species of fungi, and 22 phyla and 719 species of archaea were identified.

**Fig. 2:**
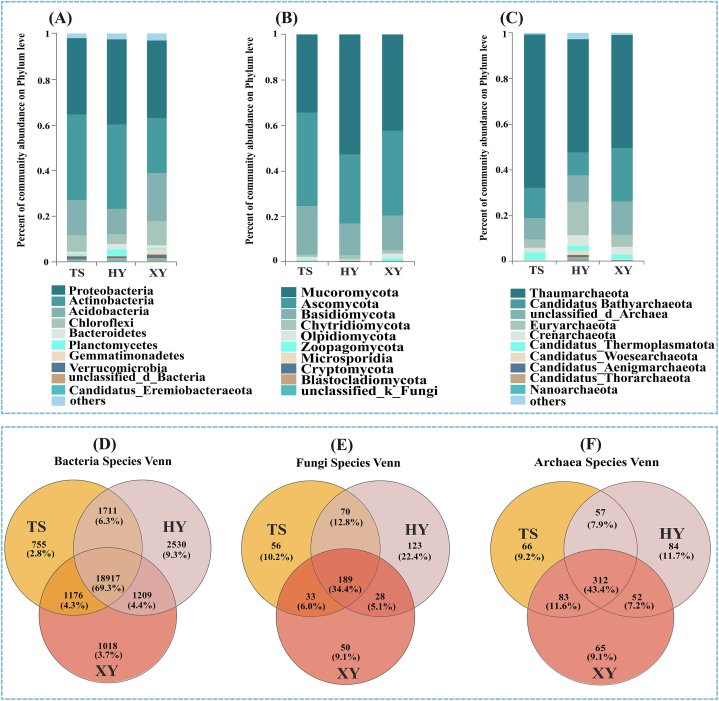
Composition of soil microbial communities in tea plantations under different modes of agricultural management; (A) bar diagrams of the top 10 bacteria community abundances at phylum level; (B) bar diagrams of the top 10 fungi community abundances at phylum level; (C) bar diagrams of the top 10 archaea community abundances at phylum level; (D) species-level based Venn diagrams for bacteria; (E) Species-level based Venn diagrams for fungi; (F) Species-level based Venn diagrams for archaea.

The Venn diagram showed that at the species level, the three tea plantations had 69.3 % similar bacterial species (Fig. 2D), 34.4 % similar fungal species (Fig. 2E), and 43.3 % similar archaeal species (Fig. 2F). The three most dominant bacteria at the Phylum level were Proteobacteria, Actinobacteria, and Acidobacteria. HY soil had the highest Ascomycota (37.38 %) and TS soil had the highest Actinomycota (37.35 %). At the Phylum level, the top three dominant fungal phyla were Mucoromycota, Ascomycota, and Basidiomycota; the relative abundance of Mucoromycota was the highest in the HY soil (52.67 %), and that of Ascomycota (41.22 %) was the highest in the TS soil. The top three dominant archaeal phyla at the phylum level were Thaumarchaeota, Candidatus Bathyarchaeota, and Euryarchaeota. The relative abundances of Thaumarchaeota in the three tea plantation soils were the highest, accounting for 67.32 %, 49.73 %, and 49.59 %, respectively.

#### 3.2.2 Soil microbial community diversity

**Fig. 3:**
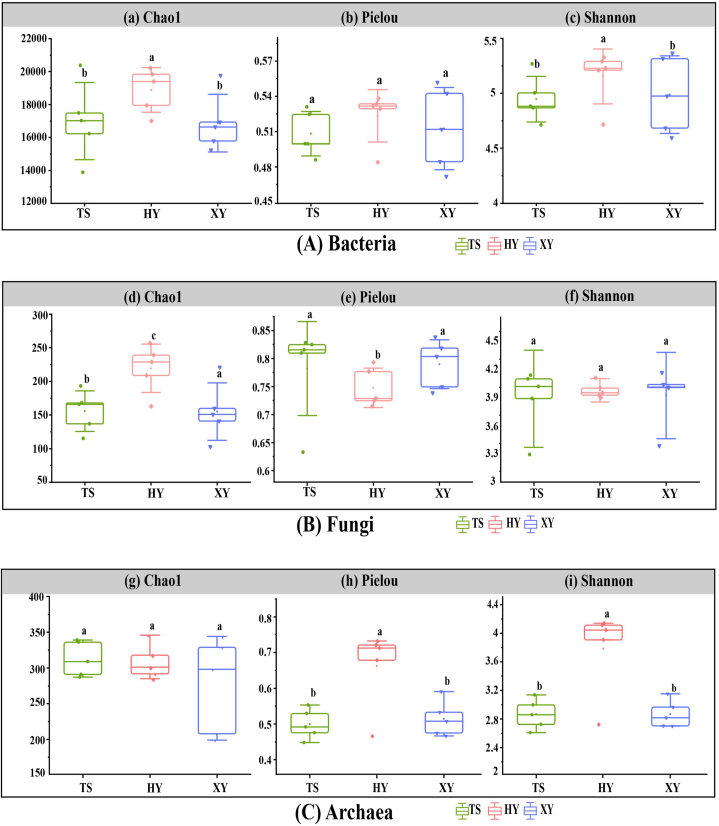
α-Diversity index of soil microbial communities in tea plantations under different agricultural management patterns.

The α diversity indices of soil microorganisms in tea plantations under different agricultural management practices are illustrated in Fig. 3. The Chao1, Shannon, and Pielou indices reflect the richness, diversity, and evenness of the microbial community, respectively. No significant differences were observed in the Pielou index of the soil microbial bacterial community, the Shannon index of the fungal community, and the Chao1 index of the archaeal community (Fig. 3) (*P* < 0.05). The Chao1, Pielou, and Shannon diversity indices of the bacterial and archaeal communities were the highest for HY. In the fungal community, the Chao1 index of HY was the highest, and the Shannon and Pielou indices (Fig. 3B) were the lowest. In summary, the richness, diversity, and evenness of the soil bacterial communities in HY were higher than those in TS and XY under Organic (Fig. 2, Fig. 3A), indicating that the soil ecosystem tended to be more stable under Organic than under PolFree and Conv.

**Fig. 4:**
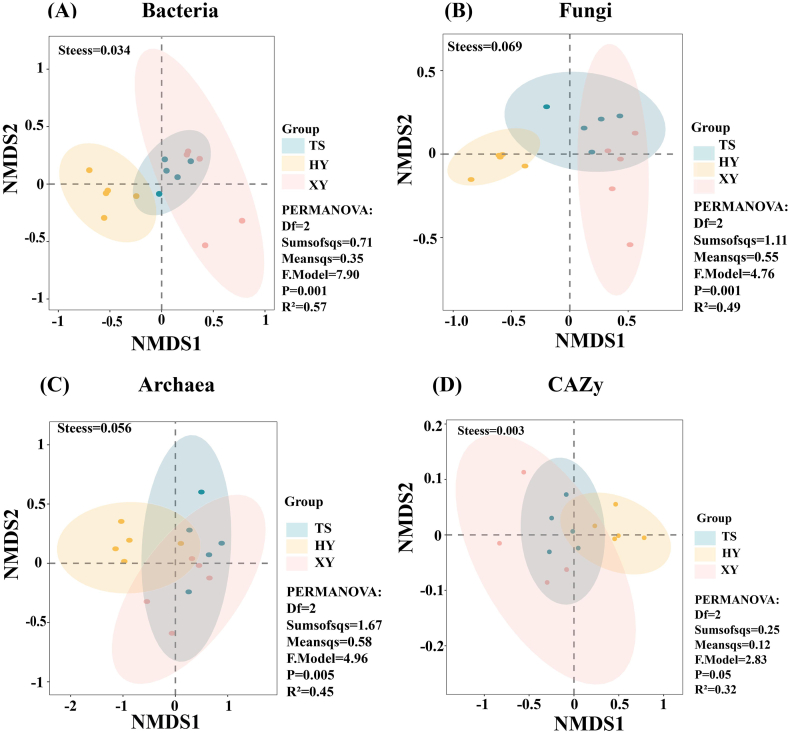
Non-metric dimensional scaling (NMDS) analysis of soil microorganisms in tea plantations under different management modes, A bacteria community; B fungi community; C archaea community; D CAZy community.

To more intuitively explore the similarities and differences in soil microorganisms in the tea plantations, NMDS analysis of the soil microbial community composition in the three tea plantations was conducted using the Bray-Curtis distance algorithm (Fig. 4). The Stress values were all <0.1, indicating that the NMDS analysis fit meets the standard and has correct explanatory significance. More overlaps and closer distances were observed between TS and XY, indicating that their microbial community structures were similar. The overlap between the HY tea plantation and the TS and XY tea plantations was smaller and farther apart, indicating that the soil microbial community compositions of HY and the other two tea plantations were less similar in structure. The results of PERMANOVA analysis showed that bacterial (*P* = 0.001, R^2^ = 0.57), fungal (*P* = 0.001, R^2^ = 0.49) and archaeal (*P* = 0.005, R^2^ = 0.45) communities differed significantly across different modes of agricultural management.

### 3.3 Correlation analysis of soil physicochemical properties and tea quality with soil microbial community structure, respectively

**Fig. 5:**
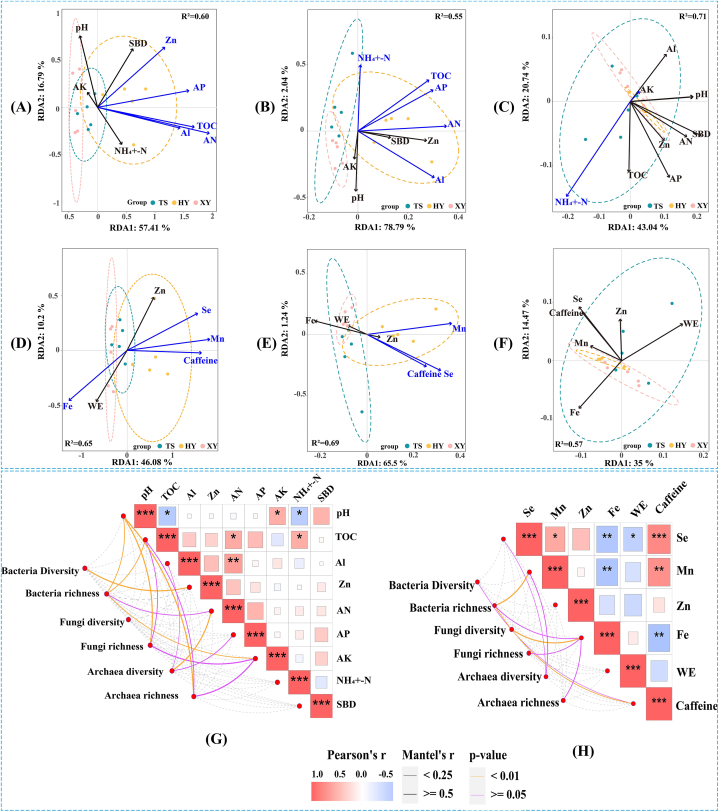
RDA and Mantle test analysis of soil microbial communities with soil physicochemical properties and tea quality, respectively (VIF <5). (A), (B), and (C) are RDA of bacteria, fungi, and archaea communities with soil physicochemical properties, respectively; (D), (E), and (F) are RDA of bacteria, fungi, and archaea communities with tea quality, respectively; blue lines represent significant correlation; black represents no correlation (*P* < 0.05). (G), (H) Mantel test analysis of microbial community diversity and abundance regarding soil physicochemical properties, respectively. (For interpretation of the references to colour in this figure legend, the reader is referred to the Web version of this article.)

The results of RDA analysis showed that the soil physicochemical factors after screening (VIF <5) explained 74.2 %, 80.83 %, and 63.78 % of the variation in bacterial, fungal, and archaeal communities, respectively (Fig. 5A, B, and C; Table S3). Tea plantation soil AN, TOC, AP, and AL were the main influencing factors of bacterial and fungal communities, and all of them pointed to HY, indicating a significant influence on bacterial and fungal communities in Organic tea plantations (*P* < 0.05). NH_4_^+^-N was the main influencing factor of the archaeal community and pointed to XY, indicating a higher influence on PolFree tea plantations (*P* < 0.05). The Mantel test analysis showed that soil Zn, pH, TOC, and AK were correlated with bacterial diversity and richness (Fig. 5G, Table S2); soil pH, TOC, and AK were correlated with fungal richness; and soil Al, AN, AP, pH, TOC, and AK were correlated with archaea diversity and richness (*P* < 0.05).

The results of RDA analysis showed that the tea quality indicators after screening (VIF <10) explained 56.28 %, 66.74 %, and 49.74 % of the variation in bacterial, fungal, and archaeal communities, respectively. Se, Mn, and caffeine in the tea garden soils were the major influencing factors of their bacterial and fungal communities. Additionally, they all pointed to HY, indicating a high influence on organic tea plantations (*P* < 0.05). None of the tea indicators were significantly correlated with the archaeal community. Mantel test analysis results showed (Fig. 5H, Table S2) that tea Mn, Fe, and caffeine were correlated with bacterial diversity and richness; tea Fe was correlated with fungal diversity and richness; and tea Se, Mn, Fe, and AK were correlated with archaea diversity and richness (*P* < 0.05).

### 3.4 Molecular ecological network analysis of soil microbial community and function in tea plantations with different agricultural management patterns

**Fig. 6:**
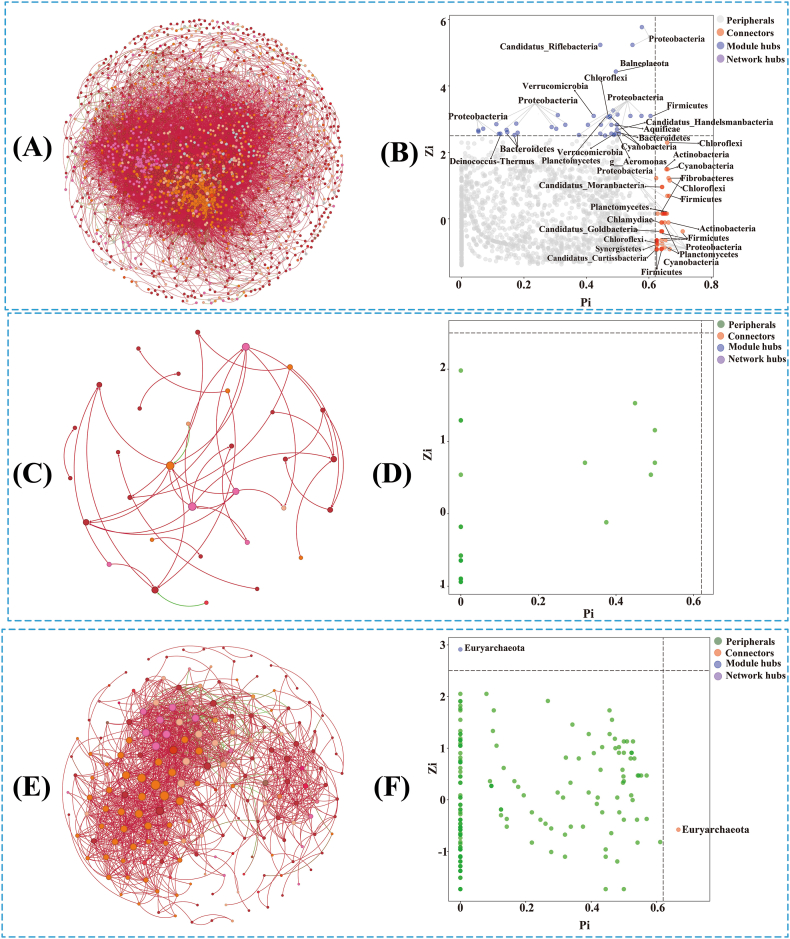
Zi-Pi and network visualization analysis of soil microbial community in tea plantations. (A)–(B), bacterial community network analysis and Zi-Pi; (C)–(D), fungi community network analysis and Zi-Pi analysis; (E)–(F), archaea community network analysis and Zi-P analysis.

In this study, the soil microbial network of tea plantations was divided into three modules according to different agricultural management modes (Table S4). Zi-Pi diagrams were used to describe the distribution of soil microorganisms in tea plantations with different agricultural management modes (Fig. 6). The keystone species of the connectors, module hubs, and network hubs are marked in the corresponding Zi-Pi plots. At the phylum level, many nodes (>90 %) in the three tea plantation soil microbial network maps were classified as peripherals, indicating that no highly connected nodes (peripherals) existed within and between modules, and no network hubs appeared. The remaining key species were found mainly in Module hubs (Zi > 2.5, Pi < 0.62) and Connectors (Zi < 2.5, Pi > 0.62). The top five species in frequency of occurrence for bacteria were Proteobacteria, Firmicutes, Planctomycetes, Cyanobacteria, and Chloroflexi (Fig. 6B); the key archaea species was Euryarchaeota (Fig. 6F), and there were no obvious fungal species (Fig. 6D). The network analysis showed that the network topology values of the bacterial community were all higher than those of fungi and archaea; therefore, the network structure of bacteria was the most complex (Fig. 6 and Table S4). In all the tree plantations, the number of positive sides of soil for microorganisms was significantly higher than the number of negative sides (Fig. 6 and Table S4). The number of microbial nodes, diameter, and average paths were higher in the HY soil than in the TS and XY soils. However, bacterial and archaeal average degrees in the HY soil were lower than those of TS and HY, and the differences in other network topology values were not significant. Overall, the results suggest that the soil structure in tea plantations is complex and, therefore, has potential self-healing capacity. The number of nodes, diameter, and average paths in the tea plantation in the Organic agricultural management treatments was considerably high; however, the average degree was relatively low, indicating that the soil microbial network structure of the tea plantation in the organic agricultural management treatment is more stable and complex.

### 3.5 Analysis of CAZy functional genes of soil microorganisms in tea plantations with different agricultural management patterns

The CAZy database (http://www.cazy.org/) comprises six carbohydrate-active enzyme families: glycoside hydrolases (GHs), glycosyl transferases (GTs), polysaccharide lyases (PLs), carbohydrate esterases (CEs), auxiliary activities (AAs), and carbohydrate-binding modules (CBMs). The six carbohydrate-active enzymes were present in the three tea plantations, and their relative abundances changed slightly; however, the proportions of the six carbohydrate-active enzymes changed significantly, with a trend of GHs > GTs > CEs > AAs > CBMs > PLs, with GH and GT genes being dominant (*P* < 0.05). The CAZy genes in the HY significantly differed from those in the TS and XY, and the relative abundance of CAZy genes was significantly higher in the HY than in the TS and XY (Table 2).

**Table 2:** Number of CAZy (Class) genes for different agricultural management models.

CAZy	TS	HY	XY
GHs	142362.80 ± 28647.82b	300726.80 ± 73218.55a	104405.60 ± 35408.55b
GTs	126285.20 ± 21675.175b	267211.60 ± 66454.49a	105381 ± 38567.309b
CEs	65543.20 ± 10128.66b	132552.00 ± 32128.88a	50201.20 ± 17014.26b
AAs	37992.80 ± 44267.13b	67415.20 ± 13049.68a	29411.20 ± 10216.27b
CBMs	9112.80 ± 1573.37b	16004.40 ± 3179.31a	6792.00 ± 1911.541b
PLs	5938.00 ± 1030.22b	11594.00 ± 3777.00a	6456.00 ± 3164.74b

*Values represent means ± standard errors (n = 5). Different lowercase letters indicate significant differences between tea plantations with different agricultural management patterns (*P* < 0.05).

#### 3.5.1 Contribution analysis of soil microorganisms to CAZy functional genes

**Fig. 7:**
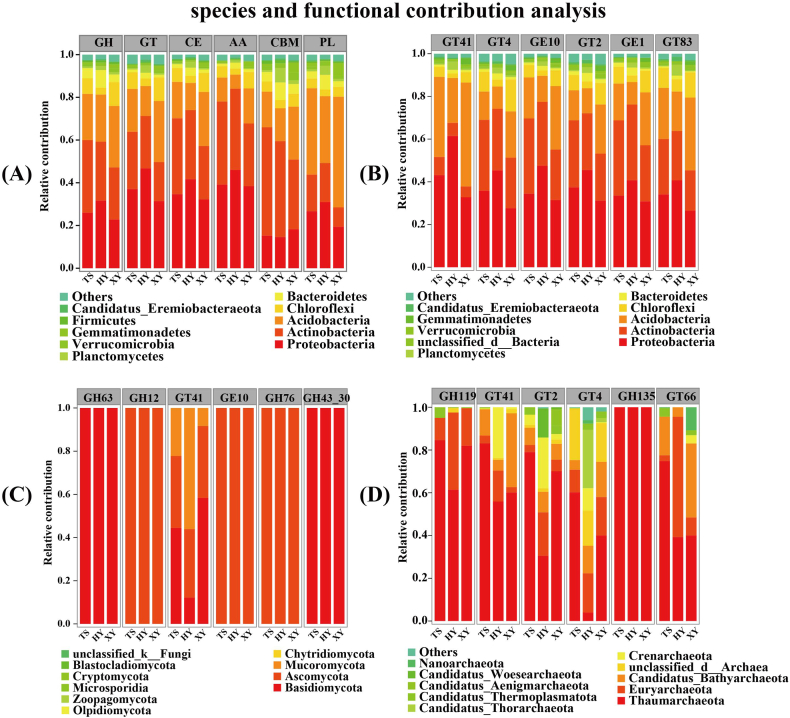
Plot of the contribution of the top 10 dominant species (phylum) of microbial community abundance to CAZy genes in tea plantations with different agricultural management patterns; (A) Contribution of total soil microbial community (phylum) to CAZy genes; (B) Contribution of bacteria community to CAZy genes; (C) Contribution of fungi community to CAZy genes; (D) Contribution of archaea community to CAZy genes.

CAZy genes, at the class level, were analysed to study the relationships between CAZy genes and soil microbial community structure; the major groups of functional genes were all associated with bacterial communities, with high proportions of Proteobacteria (46.76–14.63 %), Actinobacteria (50.77–9.07 %), Acidobacteria (51.83–6.60 %), and Chloroflexi (11.05–3.06 %). The proportions of the major functional genes in the HY soil were greater than those in the TS and XY soils (Fig. 7A). An analysis of the contribution between CAZy genes and species at the family level demonstrated that the functional genes of GT41, GT4, GE10, GT2, GE1, and GT83 in bacteria communities mainly influenced Proteobacteria (46.43–14.71 %), and the proportional values in the HY soil were higher than those in the TS and XY soils (Fig. 7B). The main functional gene contributors in fungi communities were Basidiomycota, Ascomycota, and Mucoromycota, with Basidiomycota (100 %) as the main contributor to GH43_30, and Ascomycota as the main contributor to CH12, CE10, and GH76 (Fig. 7C). The main functional gene contributors in archaeal communities were Thaumarchaeota (3.94–100 %) and Euryarchaeota (2.53–56.81 %) (Fig. 7D).

#### 3.5.2 Correlation analysis between CAZy functional genes, soil physicochemical properties and tea quality, respectively

**Fig. 8:**
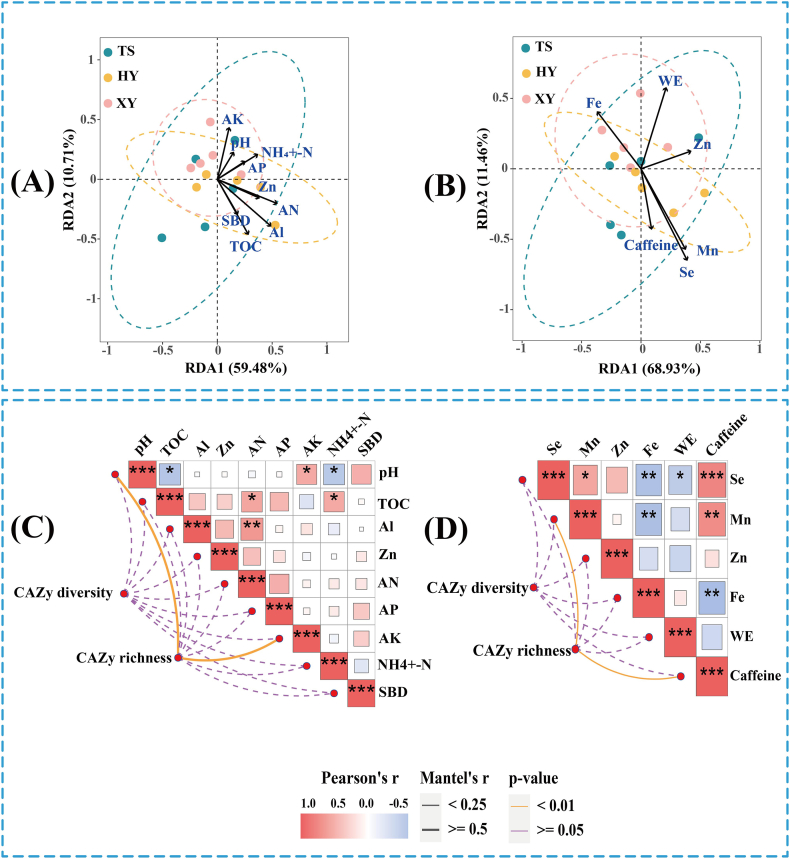
RDA and Mantle test analysis of CAZy genes with soil physicochemical properties and tea quality, respectively. (A) is RDA of CAZy genes with soil physicochemical properties. (B) is RDA of CAZy genes with tea quality. (C) is Mantle test analysis of CAZy genes with soil physicochemical properties. (D) is Mantle test analysis of CAZy genes with tea quality (VIF <5).

The Stress values were all <0.1, indicating that the NMDS analysis fit fulfilled the standard and had correct explanatory significance. The NMDS results revealed that the soil CAZy gene compositions in samples from different agricultural management treatments differed significantly. Therefore, agricultural management practice influenced functional gene composition significantly (*R*^2^ = 0.32, *P* = 0.05, F value = 2.83) (Fig. 4D). The RDA demonstrated that 60.58 % and 74.06 % of the soil physicochemical property and tea quality indicator variables were explained, respectively; therefore, tea quality indicators had a great effect on CAZy gene composition (Fig. 8A). Mantel test analysis showed that pH, Se, WC, TN, and AK, under soil physicochemical properties, and Mn and caffeine, under tea quality indicators, were the dominant factors influencing CAZy gene richness (Fig. 7C and D and Table S5).

**Fig. 9:**
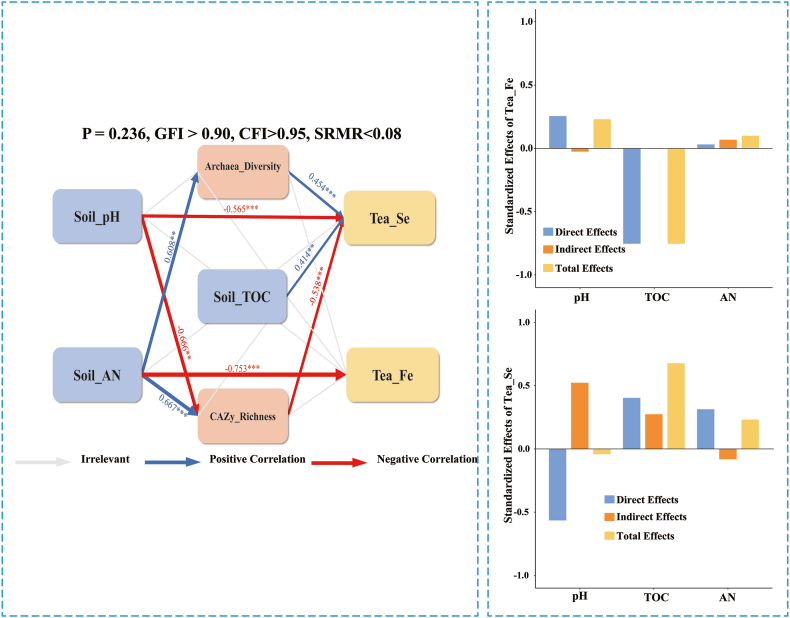
Structural equation modelling (SEM) between soil microbial community, soil physicochemical properties, and tea quality in tea plantations under different agricultural management patterns (*P* = 0.236, CFI>0.90, GFI >0.95, SRMR <0.08).

The best fit SEM was used to further explain the direct or indirect effects of soil physicochemical properties and soil microbial diversity on tea quality to obtain the key factors influencing tea quality (*P* = 0.236, CIF/GIF >0.95, SRMR <0.08). Soil physicochemical properties and microbial community diversity had strong direct and indirect effects on tea quality (Fig. 9). Soil pH and TOC affected tea Se, and soil AN directly affected tea Fe. TOC and tea Se were positively correlated. pH and AN were negatively correlated with tea quality. Soil AN also indirectly affected tea Se by significantly influencing microbial diversity.

## 4 Discussion

### 4.1 Effects of different agricultural management patterns on microorganisms

Soil microorganisms with a high frequency of occurrence in the study area were Proteobacteria, Firmicutes, Planctomycetes, Cyanobacteria, Chloroflexi, and Euryarchaeota (Zi > 2.5 and Pi < 0.62; Zi < 2.5 and Pi > 0.62), which is consistent with the findings of Janssen [46]. However, there were substantial differences in soil microbial community structure in tea plantations with different agricultural management patterns. The results of the microbial ecological network analysis of tea plantation soils showed that eutrophication bacterial groups (Proteobacteria, 30.2 %) and major bacterial decomposers of organic matter (Actinobacteria, 38.98 %), as the keystone species, had high total relative abundances in the soil of Organic tea plantations. Acidobacteria (oligotrophic group of bacteria) were less represented in Organic tea plantation soils (11.05 %), and Chloroflexi, which contributes to soil acidification, is a keystone clade present only in Conv tea plantation soil [47]. This is possibly due to the organic management pattern of adopting organic fertilisers to fulfil the nutritional and growth needs of crops through rich crop rotations [47]. Additionally, the eutrophication bacterial population is closely associated with the large amount of available nutrients in the soil [33], which explains the high levels of microbial diversity and superior community structure in soils in organic tea plantations [17,34,48]. Additionally, key microorganisms involved in soil C and nitrogen cycling and plant interactions, such as Thaumashaea [49] and Euryarchaeota [50], accounted for relatively high proportions of the abundance of microorganisms in organic tea plantation soil. Therefore, the results of this study revealed that the organic management practice (pattern) of applying livestock manure and organic fertiliser for rapeseed enables the soil to establish many interactive microecological communities and enhances microbial function, making its soil ecosystem multifunctionality superior to those of the other two management practices.

### 4.2 Effects of different agricultural management patterns on soil physicochemical properties and tea quality

In our study, soil Se, K, Zn, and Al contents under organic tea plantations were higher than those under the other two agricultural management practices. The quality of tea can be attributed to the direct and indirect effects of soil physicochemical properties and beneficial micronutrients within certain limits [51,52]. Tea quality had strong direct and indirect correlations with soil microbial community structure and soil physicochemical properties through SEM, respectively. Soil physicochemical properties comprehensively reflect soil fertility, and changes in any of the soil physicochemical indicators influence other components of the soil ecosystem [53]. Gu [22] reported that long-term organic fertiliser application enhanced the resistance of soil microorganisms to stress in tea gardens and influenced tea quality [54]. The results of our study also demonstrate that organic agricultural management can improve soil fertility in tea plantations, optimise soil microbial community composition and structure, promote many table and complex soil ecosystems, and thus improve tea leaf quality. The findings of this study enhance the understanding of soil ecosystems in tea plantations under different agricultural management practices and provide a scientific reference for the sustainable improvement of soil fertility and tea quality.

### 4.3 Effects of different agricultural management patterns on CAZy functional genes

Agricultural management measures (tillage, fertilisation) influence soil physicochemical properties and soil microbial community structure [3,8], affecting C cycling processes mediated by soil microbes [18]. The relative abundances of soil CAZy genes in organic tea plantations were significantly higher than those in tea plantations that used other management practices (PolFre and Conv), which can be attributed to the C pool of the farming system being the most active one in the entire terrestrial ecosystem, and different agricultural management practices can influence the C cycle [55]. Soil CAZy genes, as key genes involved in soil C cycling, are essential for the maintenance and development of soil ecosystems [56,57]. Therefore, organically managed tea plantation soil ecosystems are many stable. Bacteria were the major microbial community influencing CAZy genes, with the percentage of dominant species in organic tea gardens being higher than those in the other two agricultural management patterns. This result to bacteria, key decomposers of various organic substances, playing a key role in the soil C cycle [58].

Soil nutrients provide a source of energy for soil microbial activities, and microbial community structure and diversity affect CAZy gene structure and diversity [59]. The findings of this study demonstrate that soil microbes in tea plantations under organic management are many active in decomposing residues and have many potential functions in the soil C cycle [60], which can improve soil fertility, influencing tea leaf quality and yield [61].

## 5 Conclusions

This study showed close relationships among soil microorganisms, soil fertility, and tea quality. The Organic management pattern enhanced soil microbial community diversity, optimised community structure, and increased the relative abundance of CAZy functional genes in the soil, suggesting that soil microorganisms in organic tea plantations exhibit stronger residue decomposition activity and significant potential functions in the soil C cycle, improving tea plantation soil quality. The SEM results also showed strong direct and indirect relationships between soil physicochemical properties, soil microbes, and tea quality. For example, soil physicochemical properties (pH, TOC, and AN) directly and significantly affected tea Se and Fe, or indirectly affected tea Se and Fe by influencing soil microbes (archaeal community diversity and CAZy abundance); therefore, soil physicochemical indicators and soil microbes can be used as crucial bio-indicators for evaluating tea quality. The findings of this study enhance the understanding of soil microbial ecosystems in tea plantations and provide novel perspectives that can facilitate sustainable modern agricultural development in ecologically fragile karst areas.

## Funding

This study was supported by the 10.13039/501100001809National Natural Science Foundation of China (32260120), the World Top Discipline Program of Guizhou Province: Karst Ecoenvironment Sciences (No.125 2019 Qianjiao Keyan Fa), the 10.13039/501100009619Science and Technology Project of Guiyang City ([2020]718), the 10.13039/501100013055Innovation Group Project of Education Department of Guizhou Province ([2021]013) and the 10.13039/501100005329Natural Science Foundation of Guizhou Province (Qiankehejichu-ZK [2023] General 257), the Training Program for High-level Innovative Talents of Guizhou Province (Qiankehepingtairencai-GCC [2023]032).

## Data availability

All raw data generated in this study have been uploaded to the NCBI BioProject database under accession number PRJNA972911. All the data analysis results obtained during this study are included in the manuscript and its Supplementary Information.

## Ethics statement

Review and/or approval by an ethics committee was not needed for this study, because did not include any human or animal studies.

## CRediT authorship contribution statement

**Tianyi Pu:** Writing – review & editing, Writing – original draft, Visualization, Validation, Software, Methodology, Investigation, Formal analysis, Data curation, Conceptualization. **Ni Zhang:** Writing – review & editing, Writing – original draft, Visualization, Validation, Software, Methodology, Data curation, Conceptualization. **Jinqiu Wang:** Writing – review & editing, Writing – original draft, Visualization, Software, Methodology, Formal analysis, Data curation, Conceptualization. **Zhibing Zhao:** Writing – review & editing, Writing – original draft, Validation, Formal analysis, Data curation, Conceptualization. **Weiwen Tan:** Writing – review & editing, Writing – original draft, Software, Formal analysis, Data curation, Conceptualization. **Can Li:** Writing – review & editing, Writing – original draft, Visualization, Validation, Software, Project administration. **Yuehua Song:** Writing – review & editing, Writing – original draft, Visualization, Validation, Supervision, Investigation, Funding acquisition, Conceptualization.

## Declaration of competing interest

This study was supported by the 10.13039/501100001809National Natural Science Foundation of China (32260120), the Training Program for High-level Innovative Talents of Guizhou Province (Qiankehepingtairencai-GCC [2023]032), the 10.13039/501100005329Natural Science Foundation of Guizhou Province (Qiankehejichu-ZK [2023] General 257), and the Science and Technology Innovation Talent Team Building Project of Guizhou Province (Qiankehepingtairencai-CXTD [2023]010).
