# Supplementary material for: Organic management pattern improves microbial community diversity and alters microbial network structure in karst tea plantation
Source: Heliyon. 2024 May 19;10(10):e31528. doi: 10.1016/j.heliyon.2024.e31528 (PMC11141352; doi:10.1016/j.heliyon.2024.e31528)
Supplement: Multimedia component 6 [file mmc6.docx]

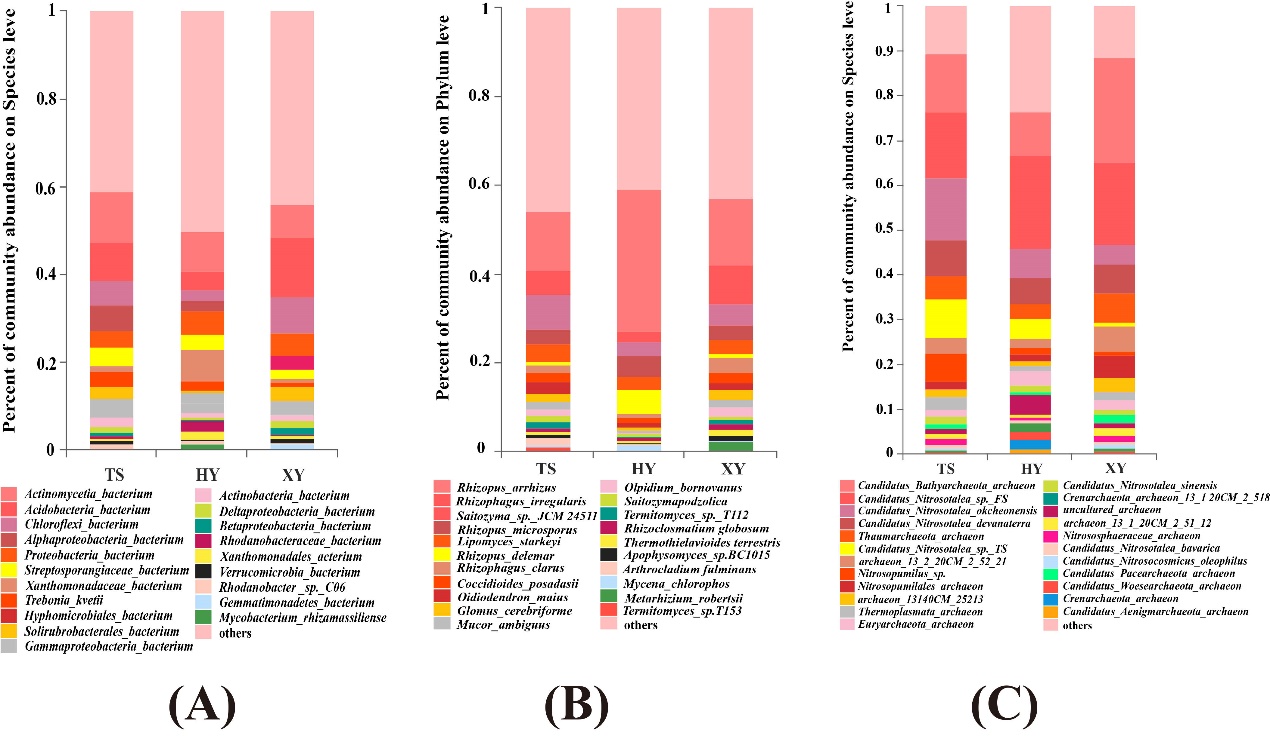


**Figure S1:** Composition of soil microbial communities in tea plantations under different modes of agricultural management, species-level based community structure diagrams; (A) a bar chart of the top 20 abundances of bacteria based on species level; (B) a bar chart of the top 20 abundances of fungi based on species level; (c) a bar chart of the top 20 abundances of archaea based on species level
