# Supplementary material for: Organic management pattern improves microbial community diversity and alters microbial network structure in karst tea plantation
Source: Heliyon. 2024 May 19;10(10):e31528. doi: 10.1016/j.heliyon.2024.e31528 (PMC11141352; doi:10.1016/j.heliyon.2024.e31528)
Supplement: Multimedia component 7 [file mmc7.docx]

1 **Determination of physical and chemical properties of soils**

The air-dried samples to be measured were mixed thoroughly, reduced to 100 g according to the tetrad method, and crushed. The seeds were sieved through a 40-mesh sieve and stored in sample bottles for subsequent use. **Soil metal elements (Zn, K)** were determined directly using ICP-OES (iCAP-7200, Thermo Fisher Scientific, MA, USA). The preparation of the sample solution was as follows: 0.2 g of the dry sample was placed in a conical flask, and 5 ml nitric acid and 1 ml perchloric acid were added; the flask was placed on a graphite ablator at 200 ℃ until the solution became colourless and transparent (approximately 2–3 h, during which hydrogen peroxide can be added dropwise); the contents of the flask was poured into a 50 ml volumetric flask; and the volume was set to the scale with 1% HNO3, and this is the extraction solution. The final decoction extracts were determined directly by ICP-OES.

**Soil Se** determination was conducted using atomic fluorescence spectrometry (AFS), and the air-dried soil samples were divided into four parts, crushed, sieved through a 0.149 mm aperture sieve, and mixed into sample bottles for subsequent experiments. Two grams of the sample to be tested was placed in a 100 m triangular flask, and 10-15 mL perchloric acid mixture was added; the flask was then covered with a small funnel and left overnight. The next day, in a 160 ℃ automatic temperature-controlled digestion furnace, digestion to colourless (soil samples into grey-white) was performed. This process was continued until white smoke was observed. 1 min-2 min to remove the cooling, and then to the triangular vials, add 10 mL, hydrochloric acid solution. Next, the triangular flask was placed in a boiling water bath, heated for 10 min, removed, and cooled to room temperature. Deionised water was digested in a 50 mL volumetric flask, and the volume was fixed to the graduation, shaking well. The test solution was retained for testing. The fluorescence signals of the test solutions were measured under the same conditions as the selenium standard series. Next, 10–20 mL of the reduced and calibrated solutions was used to determine the peak fluorescence signals of the test solutions.

**2 Determination of Tea Quality Properties**

The spectrophotometric method was used to determine the **tea polyphenols (GTP)** in tea. The preparation of the test solution was as follows: an appropriate amount of the well-mixed sample was weighed and placed in a centrifuge tube; 5 mL of 70% methanol aqueous solution was added; the mixture was preheated at 70 ℃, stirred well with a glass rod, immediately transferred to a 70 ℃ water bath, extract for 10 min (stirring every 5 min), removed and cooled to room temperature, and centrifuged at 4000 r/min for 10 min. The supernatant was transferred to a 10 mL volumetric flask. The residue was re-extracted once with 5 mL 70% methanol aqueous solution, and the combined extracts were fixed to 50 mL, shaken well, passed through a 0.45 μm filter membrane, and measured. The preparation of standard curves was as follows: for the gallic acid working solution, 1, 2, 3, 4, and 5 of 100 mg/L gallic acid standard reserve solution was pipetted into a 100 mL volumetric flask, respectively; the volume was adjusted to the scale with water, and shaken well; and the concentration was 1, 2, 3, 4, and 5, respectively. Next, using a pipette, 0.1 mL each of gallic acid working solution, water, and test solution was placed into a graduated test tube; Folinol (10%; 5 ml) was added into each test tube and shaken well. Within 3-8 min of the reaction, 4 mL of 7.5% sodium carbonate solution was added. Water was added to the gradient and shaken well, it was left at room temperature for 60 min, and the absorbance was measured spectrophotometrically at 760 nm by using a 10 mm cuvette. According to the absorbance of the gallic acid working solution and the concentration of each working solution, the standard curve was calculated.

**Water extracts (WE)** of tea were determined using the drying method. A clean glass weighing dish containing WE was placed in a 101–105 ℃ drying oven for 1.0 h; removed; placed in a desiccator for cooling for 0.5 h; weighed; and repeated until the drying of the front and back of the two masses had a difference of no more than 2 mg, that is, the constant weight. Next, the appropriate amount of well-mixed specimen was placed in a conical flask and weighed. Boiling distilled water (300 mL) was added to the conical flask, which was immediately transferred to a boiling water bath immersion for 45 min while shaking it from time to time. Immediately after the extraction was completed, filtration under reduced pressure while hot was conducted. Approximately 150 mL boiling distilled water was used to wash the residue several times. Place the residue and filter paper together in a constant weight weighing tray, cover with filter paper and weigh, then dry in an oven at 120 °C. The cap was obliquely supported on the bottle side. The duration of drying was 2–4 h. It was covered, removed, and cooled in a desiccator for 0.5 h after weighing. Next, it was dried in a 120 ℃ drying oven for approximately 1 h, removed, and cooled in a desiccator for 0.5 h before weighing. Repeat the above operation until the difference in mass between the two times before and after is not more than 2mg, which is the constant weight.

**Caffeine** was determined using an Agilent C18 column (250 × 4.6 mm, 5 µm), with methanol as the A phase and water as the B phase.; a flow rate of 1 ml/min for 12 min, a detection wavelength of 271 nm, a column temperature of 25 ℃, and an injection volume of 10 μl were used. An appropriate amount of the sample was collected and mixed with 70% methanol (ACS, USA) and vortexed. Next, it was placed in a boiling water bath for 30 min until the colour of the extract was light. The heating was immediately stopped when it cooled and was then siphoned off. Subsequently, 70% methanol was added to 25 mL, which was passed through a 0.45 μm microporous filter membrane, and liquid chromatography was used for detection.

An inductively coupled plasma mass spectrometer (iCAPQ, Thermo, USA) was used to determine the **metal elements (Fe, Mn, Zn) in tea**. First, the appropriate amount of the sample was weighed and placed into a polytetrafluoroethylene ablation jar, to which 5 mL nitric acid was added. After the reaction was finished, a lid was placed on the jar, and the jar was placed into a microwave ablator (temperature: 100, 140, 160, 180, and 190 ℃; holding time: 3, 3, 3, 3, 3, and 15 min). The temperature was cooled to less than 50 ℃. Next, the jar was removed from the ablator and placed into a fume cupboard. The ablator was opened, and it was wetted and washed with ultrapure water and transferred to a 25 mL flask. The volume flask was rinsed at least 3~4 times, and the volume was diluted and fixed with ultrapure water to the scale to be measured (ICP-MS instrument parameters: RF power, 1550 W; pump speed, 40 rpm; , S/C temperature, 2.7 ℃; smpl depth, 5 mm; cool flow, 14 l/min; auxilliary flow, 0.8l/min; auxilliary flow, 0.8l/min; auxilliary flow, 0.8l/min; auxilliary flow, 0.8l/min; auxilliary flow,0.8l/min; auxilliary flow, 0.8l/min); auxilliary flow, 0.8 l/min, nebuliser flow, 1.122l/min).
